# Feature selection to classify lameness using a smartphone-based inertial measurement unit

**DOI:** 10.1371/journal.pone.0258067

**Published:** 2021-09-30

**Authors:** Satoshi Arita, Daisuke Nishiyama, Takaya Taniguchi, Daisuke Fukui, Manabu Yamanaka, Hiroshi Yamada

**Affiliations:** Department of Orthopedic Surgery, Wakayama Medical University, Wakayama, Japan; University of Illinois, UNITED STATES

## Abstract

**Background and objectives:**

Gait can be severely affected by pain, muscle weakness, and aging resulting in lameness. Despite the high incidence of lameness, there are no studies on the features that are useful for classifying lameness patterns. Therefore, we aimed to identify features of high importance for classifying population differences in lameness patterns using an inertial measurement unit mounted above the sacral region.

**Methods:**

Features computed exhaustively for multidimensional time series consisting of three-axis angular velocities and three-axis acceleration were carefully selected using the Benjamini–Yekutieli procedure, and multiclass classification was performed using LightGBM (Microsoft Corp., Redmond, WA, USA). We calculated the relative importance of the features that contributed to the classification task in machine learning.

**Results:**

The most important feature was found to be the absolute value of the Fourier coefficients of the second frequency calculated by the one-dimensional discrete Fourier transform for real input. This was determined by the fast Fourier transformation algorithm using data of a single gait cycle of the yaw angular velocity of the pelvic region.

**Conclusions:**

Using an inertial measurement unit worn over the sacral region, we determined a set of features of high importance for classifying differences in lameness patterns based on different factors. This completely new set of indicators can be used to advance the understanding of lameness.

## Introduction

Gait is a complex movement, in which the body moves forward using the friction between the sole and the ground as support, and the centroid position moves up, down, left, and right. It is one of the most basic human movements. The smoothness of gait is easily reduced due to pain, muscle weakness, and deformation caused by aging and various diseases. For example, antalgic gait is a special type of walking with a limp that is characterized by a very short stance phase aiming to avoid pain in weight-bearing joints [1].

Gait activity recognition, the ability to identify the state of walking from body sensor data or wearable sensor data, is one of the most actively researched fields in recent years and has attracted the attention of academic disciplines and health industries. The classification of gait patterns has a great potential, as an assessment tool for diagnosing injuries and identifying high-risk gait in the elderly. Several studies have shown that wearable inertial sensors can be used to classify gait patterns [2–5].

Concerning the features that are useful for the classification of gait patterns, Quiroz et al. showed that gravity signals provide high classification accuracy, especially for the static activities of sitting, standing, and lying down. They also reported that some features extracted from angular velocity are not as useful for classification as body acceleration but can significantly improve the accuracy of the feature set of body acceleration [6]. Fuentes et al. used nine features of accelerometers for mobile phones for online motion recognition: the standard deviation and the range of the orientation angle θ, the standard deviation and the minimum value of the forward acceleration, the standard deviation of the vertical acceleration, the standard deviation and the minimum Y values, and the standard deviation and the minimum Z values [7].

In recent years, several attempts have been made to detect minute differences in gait patterns. An automatic classification by Eskofier et al., using nine reflective markers placed on the upper and lower limbs, showed 84.7% classification accuracy for sex. Moreover, the variance of the hip flexion-extension moment and the variance of the vertical ground reaction force were selected as features. The classification accuracy of the shod/barefoot classification was 98.3%, and there were two regression features: the quadratic polynomial component of the foot sagittal plane angle and the linear polynomial component of the shank sagittal plane angle. The classification accuracy for the existence of severe patellofemoral pain was 100%, and the mean hip abduction moment was identified as the only significant factor [8]. Rezaul et al. proposed a support vector machine-based approach for classifying young/old gait patterns. Their findings suggested that histogram and Poincaré plots of minimum foot clearance data during walking may provide useful gait features that can effectively recognize young/old gait [9]. Benson et al. created a feature set consisting of three discrete features (the value at heel-strike for the anterior-posterior axis of the left side, the peak maximum for the anterior-posterior axis of the right side, and the peak minimum for the vertical axis of the left side) and five advanced features (medial-lateral axis [ML]–standard deviation, ML– 25th percentile, ML–root mean squared, ML–ratio, resultant signal–Sum of fast Fourier transformation [FFT] Coefficients [3–6]). They showed that they could classify running at their preferred speed and 25% faster than their preferred speed with high accuracy (97.23%) [10].

However, to our knowledge, there are no studies on the features that are useful for classifying lameness patterns by different factors. Our aim was to identify features of high importance for classifying population differences in lameness patterns by different factors using an inertial measurement unit (IMU) mounted above the sacral region.

## Materials and methods

### Experiments and data collection

#### Participants

This study was conducted in accordance with the Ethical Guidelines for Medical and Health Research Involving Human Subjects and the ethical standards laid down in the World Medical Association Declaration of Helsinki of 1975 and its later amendments. Written informed consent was obtained from all participants. This study has been approved by the institutional review board of Wakayama Medical University Hospital (No. 3236). Sixteen healthy volunteers (sex, eight men and eight women) participated in the study.

#### Experimental method

The participants walked on a 10-m indoor walking path under the supervision of an experienced clinical researcher, and the IMU mounted above the sacral region simultaneously measured acceleration in three axes (vertical, front-back, and side-to-side directions) and angular velocity around the three axes.

**Fig 1:**
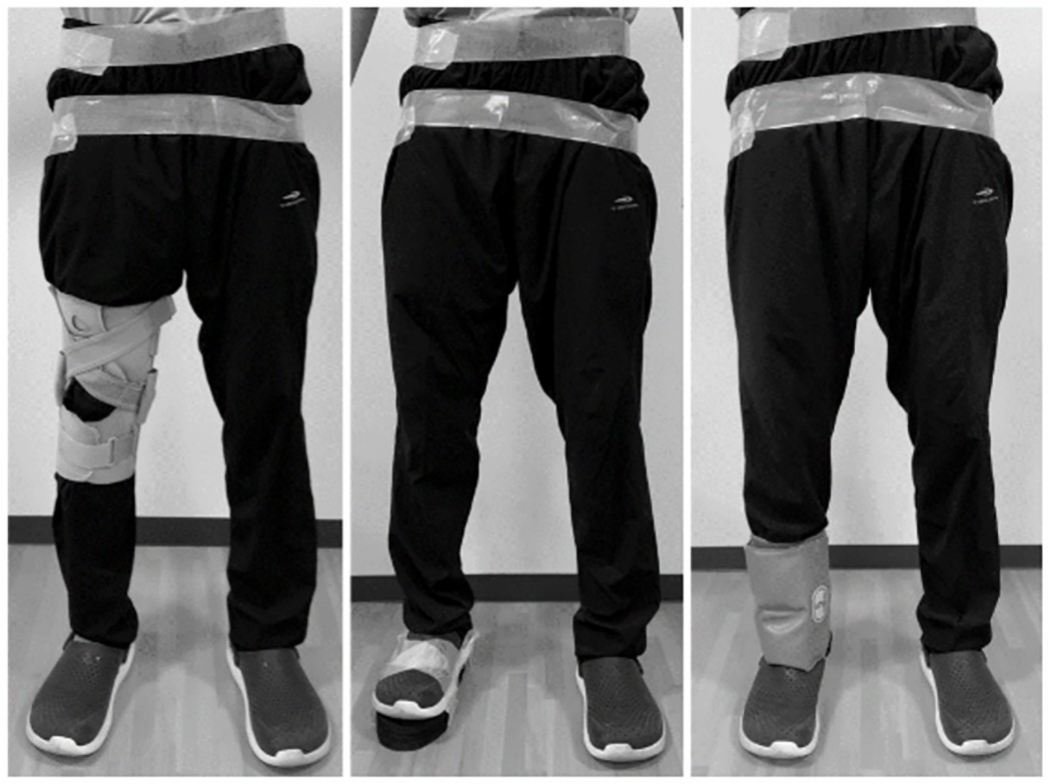
(a) The walking task was performed wearing a knee brace, (b) a shoe lift, and (c) ankle weights.

All participants in the study performed four tasks: regular gait and three simulated abnormal gaits. In the “regular gait” session, participants walked along a 10-m path, where they performed a so-called natural gait. In the simulated “abnormal gait,” the participants walked along the same path while wearing (i) a knee brace, (ii) a shoe lift, and (iii) ankle weights (Fig 1a–1c). A knee brace was used to simulate disabilities, such as knee contracture and lack of muscle coordination. The shoe lift was 3 cm thick and was used to simulate the effects of leg length discrepancy. The ankle weights were simulated for hemiplegia. To determine the optimal weight, a preliminary experiment, in which three participants (two male and one female) wore ankle weights of several different weights, was conducted before starting the walking test. The optimal weight was defined as the minimum weight at which all participants experienced difficulty while pretending to walk normally, and we selected the weight closest to 12% of their body weight from the range of weights in 0.5 increments. Participants were advised not to continue if they felt that they could not walk safely alone, and if necessary, a person walked alongside them to prevent falls. Each participant repeated the 6-s walk for 12 times in total, in three “regular gait” sessions and in three sessions of simulated “abnormal gait.” The order of the walks was randomly determined using the Microsoft Excel RANDBETWEEN function (Microsoft Corp., Redmond, WA, USA).

**Fig 2:**
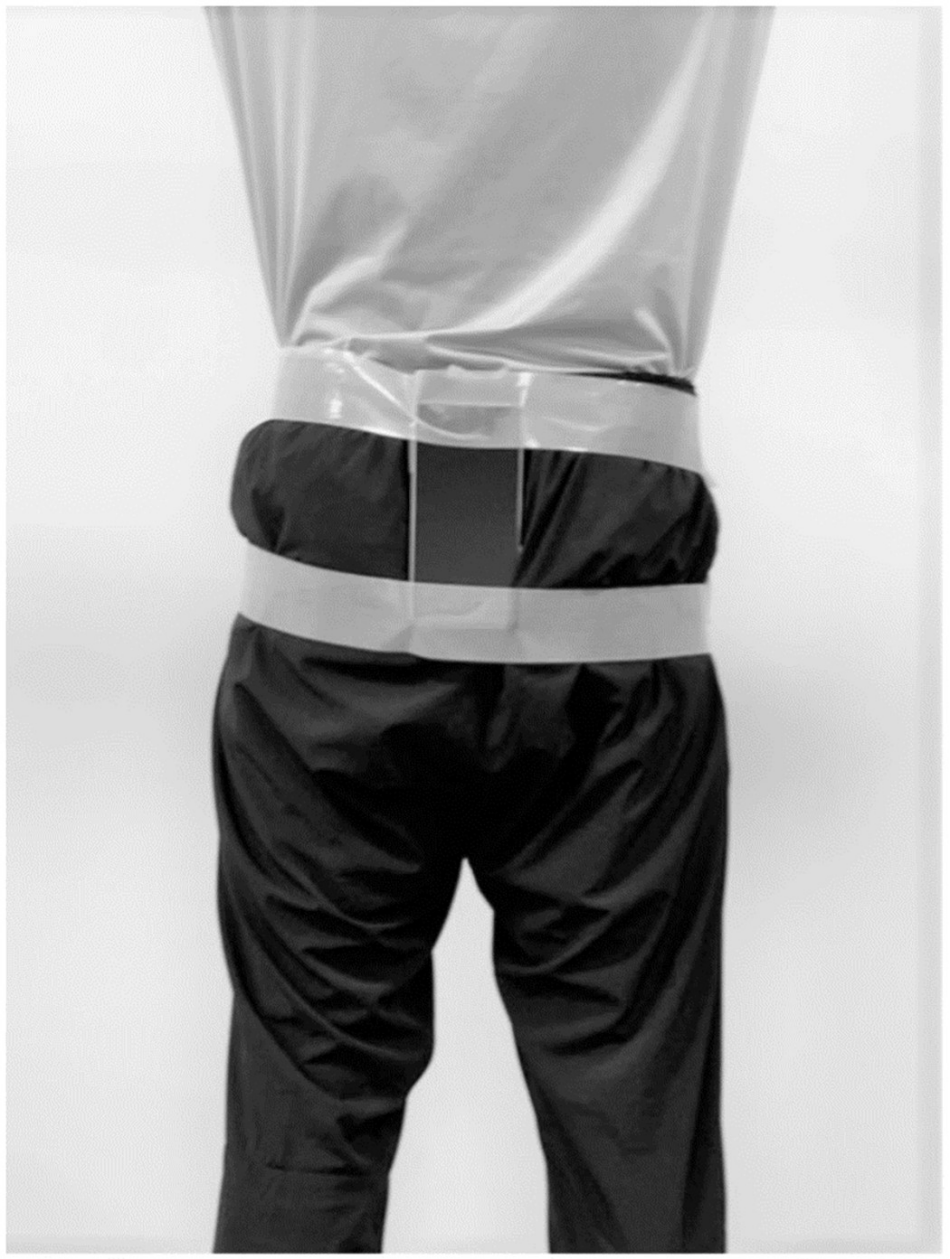
Smartphone attached to the body.

A smartphone with a built-in inertial sensor (Xperia Z5 501SO, Android 6.0.1; Sony Corp., Tokyo, Japan) was attached to the participants’ clothing at the level of the second sacral spinous process (S2), where the body’s center of gravity is located in the standing position, using a 5-cm-wide tape. The tape was fixed at the top and bottom of the smartphone and around the pelvic area (Fig 2).

We ensured that the smartphone-holding tape did not restrict the participant’s movement. The participants were briefed on the application and reviewed the audio instructions prior to the gait evaluation. The application was developed for the Android platform for use in this study to record and collect inertial sensor data using a smartphone.

With the participants in an upright position in front of the start line, the supervisor initiated the application for inertial sensor data collection. Participants performed the walking tasks while listening to rhythmic sounds at a rate of 100 beats/min and stopped walking upon hearing a beeping sound 6 s after the start sound. The data were recorded for 6 s after the start sound. Acceleration and angular velocity data were collected at sampling frequencies of 50 and 250 Hz, respectively. The collected time-series data were saved on a smartphone’s SD card in a.csv file format. The data stored on the SD card were exported to a personal computer (PC) using a USB cable, and all subsequent processing was performed using a PC.

#### Axis settings

**Fig 3:**
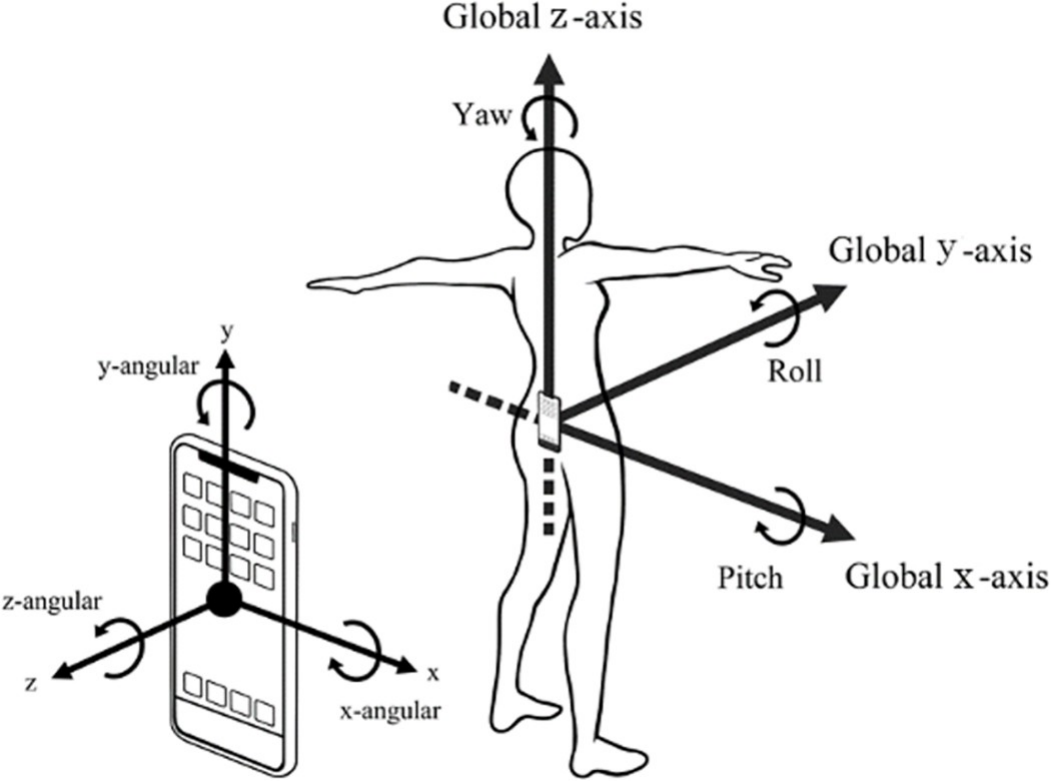
Axis settings in the device coordinate system and the global coordinate system.

The walking direction was defined as the global y-axis, the left-right direction as the global x-axis, and the vertical direction as the global z-axis (Fig 3). The accelerations obtained from the IMU were transformed from the device coordinate system to the global coordinate system, assuming that the smartphone was placed vertically with the screen facing away from the device, with the y-axis and the z-axis being the global z-axis and y-axis, respectively. The angular velocities were transformed from the device coordinate to the global coordinate system, similar to the axis transformation of acceleration.

### Data processing

The time-series data of acceleration and angular velocity obtained from the IMU were smoothed by removing high-frequency noise components of the waveform, taking the average of the values over a past fixed period using the exponentially weighted moving average (EWMA), with the weights decreasing exponentially from the most recent value. To determine the optimal moving average period, we examined several different periods using the waveforms of three randomly selected walks. In this study, the period of the smallest moving average, during which high-frequency noise >8 Hz was extinguished, was considered optimal. The angular velocity data were converted to a weighted average of the last 40 values; the acceleration data were converted to a weighted average of the last 10 values (a 10-period EWMA).

The peak of the acceleration of the vertical (global z-axis) component, which remains despite any lameness, was used as an index for clipping the gait cycle. The first to the third peak, the third to the fifth peak, and the fifth to the seventh peak of the time series in the global z-axis direction were identified as partial time series. The three gait cycles, with a window width from the first to the seventh peak, were also extracted as a partial time series. Especially, from one time series data, three data points of a single gait cycle and one data point of three gait cycles were clipped.

The acceleration and angular velocity in each direction in the same range were combined and treated as a multidimensional time series. We included 552 and 185 data points of a single and of three gait cycles, respectively, for which all multidimensional time series were complete. All signal processing was performed using Microsoft Excel (2019).

#### Feature extraction

Tsfresh is a Python package (Python Software Foundation, Wilmington, DE, USA) that is used to automatically calculate a large number of time-series characteristics or features [11]. To determine the significance of Tsfresh features for the characterization of “abnormal gait,” we conducted a comprehensive analysis of multidimensional time series for each gait cycle. We exhaustively calculated 4674 Tsfresh features from a multidimensional time series of a single gait cycle and 1104 features from a multidimensional time series of three gait cycles and defined the characteristics of each gait.

#### Feature selection

We used the “gait status” label to select Tsfresh features. The "gait status" labels were as follows: (0) regular gait, (1) wearing a knee brace, (2) wearing a shoe lift, and (3) wearing ankle weights. The *p*-value was used to determine the predictive power of each Tsfresh feature, and the Benjamini–Yekutieli procedure was used to decide which Tsfresh features to keep [12]. Multiple test procedures were performed on the training data, and the top 10 Tsfresh features were retained. We also performed a binary classification procedure between every two elements of the "gait status" labels, leaving the top 10 Tsfresh features in each combination and excluding duplicates.

#### Classification

In this study, LightGBM was used as a classifier. LightGBM is a gradient-boosting framework based on a decision-tree-based learning algorithm [13]. It is a popular boosting algorithm of fast training efficiency that can handle a large number of applications. LightGBM has been applied to electroencephalogram signal classification with some success in practical problems, such as emotion recognition [14,15] and epilepsy prediction [16]. Here, 7/8 of the training data were randomly extracted as the training (in a narrow sense) set, and the remaining (1/8) information was used as the validation set for training using the LightGBM model.

Machine learning models also generally have additional pre-specified settings called hyperparameters. Tuning the hyperparameters may help optimize performance. In this study, we used the default model with default hyperparameters and the tuned model with the hyperparameters adjusted by Optuna (Preferred Networks, Tokyo, Japan). The latter is an automatic hyperparameter optimization software framework specially designed for machine learning [17]. For the tuning of hyperparameters by Optuna, nested cross-validation (k = 3) was used, in which the training data for each fold divided by k-fold cross validation were further divided into three parts, and the optimal condition was adopted.

### Validation and evaluation metrics

The performance of each model was evaluated as the mean of 5-fold cross-validation. In the k-fold cross-validation, the original sample was randomly divided into k equally sized subsamples. Of the k subsamples, one subsample was used as test data to test the model, and the remaining k—1 subsamples were used as training data. Then, the cross-validation was repeated k times, and each of the k subsamples was used once as the test data. This validation technique rules out the possibility of the model to learn the identity of the selected feature set by ensuring that the data are not mixed into the training and test sets.

As an evaluation metric, we assessed the accuracy, which denotes the ratio of the number of correct predictions to the total number of predictions. The accuracy was calculated as follows:

$$Accuracy\;=\;\frac{TP\;+\;TN}{TP\;+\;FP\;+\;TN\;+\;FN}$$

where TP, TN, FP, and FN correspond to true positives, true negatives, false positives, and false negatives, respectively.

We calculated the relative importance of the features that contributed to the classification task in machine learning to use data from a single gait cycle and from three gait cycles. The relative importance was averaged over five cross-validation runs.

## Results

### Participant characteristics

The participants were aged between 21 and 43 years (mean age, 32.9 ± 7.7 years). The mean height and weight were 167.4 ± 8.1 cm and 62.4 ± 17.7 kg, respectively.

### Importance of features

The accuracies of the model using data from a single gait cycle and from three gait cycles were 0.871 and 0.811, respectively. It seems that the relevant features of each class were captured in the training stage, which made it possible to achieve good classification performance. The superordinate features and average relative importance for each model are listed in Tables 1 and 2.

**Table 1:** Average relative importance of the top 10 features of the model using data of a single gait cycle.

Feature names	Relative importance
**Yaw_fft_coefficient_attr_abs_coeff_2**	0.135
**Roll_fft_coefficient_attr_real_coeff_2**	0.119
**Pitch_fft_coefficient_attr_abs_coeff_1**	0.101
**Global y-axis_maximum**	0.084
**Global y-axis_quantile_q_0_8**	0.059
**Roll_fft_coefficient_attr_abs_coeff_4**	0.052
**Pitch_agg_autocorrelation_f_agg_var_maxlag_40**	0.052
**Global z-axis_quantile_q_0_2**	0.044
**Global z-axis_quantile_q_0_1**	0.038
**Yaw_change_quantiles_f_agg_var_isabs_False_qh_1_0_ql_0_8**	0.030

fft: Fast Fourier transformation; attr: Attribute; abs: Absolute; coeff: Coefficient; agg: Aggregate; var: Variance; maxlag: Maximum lag; isabs: The path is absolute (true/false); qh: Higher quantile of the corridor; ql: Lower quantile of the corridor.

**Table 2:** Average relative importance of the top 10 features of the model using data of three gait cycles.

Feature names	Relative importance
**Yaw_fft_coefficient_attr_abs_coeff_6**	0.193
**Roll_fft_coefficient_attr_real_coeff_6**	0.192
**Roll_fft_coefficient_attr_abs_coeff_12**	0.122
**Pitch_fft_coefficient_attr_abs_coeff_3**	0.069
**Global z-axis_quantile_q_0_1**	0.052
**Global y-axis_maximum**	0.044
**Pitch_sample_entropy**	0.035
**Pitch_agg_autocorrelation_f_agg_var_maxlag_40**	0.032
**Global z-axis_minimum**	0.030
**Yaw_change_quantiles_f_agg_mean_isabs_True_qh_1_0_ql_0_8**	0.027

fft: Fast Fourier transformation; attr: Attribute; abs: Absolute; coeff: Coefficient; agg: Aggregate; var: Variance; maxlag: Maximum lag; isabs: The path is absolute (true/false); qh: Higher quantile of the corridor; ql: Lower quantile of the corridor.

## Discussion

In this study, we identified important features for classifying abnormal gait using a device attached to the pelvis. The exhaustive search revealed features that have been overlooked by conventional analysis and introduced a new focus for understanding lameness. This method can be directly applied to group classification tasks.

The accuracy of the four-class classification using data from a single gait cycle was 0.871. The accuracy results of the binary classification between each type of reproduced lameness and of normal gait were excellent: 0.97 for wearing a knee brace, 0.91 for wearing a shoe lift, and 0.9 for wearing ankle weights.

The most important feature of the model using data of a single gait cycle was “Yaw_fft_coefficient_attr_abs_coeff_2.” The average relative importance of this feature was 0.135. This feature is the absolute value of the Fourier coefficient of the second frequency calculated by one-dimensional discrete Fourier transform of the pelvic yaw angular velocity data. The most important feature of the model using data of three gait cycles was “Yaw_fft_coefficient_attr_abs_coeff_6.” The average relative importance of this feature was 0.193. This feature is the absolute value of the Fourier coefficient of the sixth frequency calculated by one-dimensional discrete Fourier transform using data of three gait cycles of the yaw angular velocity of the pelvic. These features are thought to reflect the difference in the timing of the left and right peaks of the pelvic yaw angular velocity in kinematics.

The important features revealed in this study were mostly angular velocity-derived features. In the model containing data from a single gait cycle, the total relative importance of angular velocity-derived features was 0.663, and the total relative importance of acceleration-derived features was 0.326. None of the features derived from the global x-axis were included. As reported by Kavanagh et al. in their regularity analysis using approximate entropy [18], the fact that the ML direction of acceleration is the least regular variable was considered a factor.

The main limitation of this study was the fact that the abnormal gait was simulated; further, the 3-cm prosthetic height and 12% weight may have simulated a more severe lameness than that of patients. However, the proposed approach showed great potential and provided a solid foundation for further research on gait analysis. As a future project, we plan to characterize factor-related gait in patients with lameness. We would also like to conduct a large-scale study that includes data from elderly individuals to systematically analyze gait-related differences among diverse patient groups.

## Conclusions

Using an inertial measurement unit worn over the sacral region, we identified a set of features of high importance for classifying group differences in lameness patterns by different factors. This is a completely new set of indicators for understanding lameness.

## Supporting information

S1 Data (XLSX)
